# 2,2,7,7-Tetra­methyl-1,2,3,4,5,6,7,8-octa­hydro­acridine-1,8-dione

**DOI:** 10.1107/S1600536812048957

**Published:** 2012-12-15

**Authors:** Sema Öztürk Yildirim, Ray J. Butcher, Rahime Şimsek, Ahmed El-Khouly, Cihat Şafak

**Affiliations:** a Department of Chemistry, Howard University, 525 College Street NW, Washington, DC 20059, USA; b Department of Physics, Faculty of Sciences, Erciyes University, 38039 Kayseri, Turkey; c Hacettepe University, Faculty of Pharmacy, Dept. of Pharmaceutical Chemistry, 06100 Sihhiye-Ankara, Turkey

## Abstract

The whole molecule of the title compound, C_17_H_21_NO_2_, is generated by twofold rotational symmetry. The N atom and the C and H atoms in position 4 of the pyridine ring lie on the twofold axis. The cyclohexene ring has a sofa conformation with the CH_2_ C atom adjacent to the dimethyl-substituted C atom displaced by 0.5949 (16) Å from the mean plane of the other five C atoms. In the crystal, weak C—H⋯O inter­actions link the mol­ecules into chains parallel to the *a* axis. In addition, π–π stacking inter­actions [centroid–centroid distance = 3.8444 (7) Å] contribute to the stabilization of the crystal structure.

## Related literature

For background to potassium channels and biological functions and physiological roles, see: Horiuchi *et al.* (2001 ▶); Crestanello *et al.* (2000 ▶). For biological properties of 1,4-dihydro­pyridines (DHP), see: Simşek *et al.* (2004 ▶); Fincan *et al.* (2012 ▶); Gündüz *et al.* (2009 ▶); Pyrko (2008 ▶); Li *et al.* (2010 ▶). For geometric analysis, see: Cremer & Pople (1975 ▶). For a description of the Cambridge Structural Database, see: Allen (2002 ▶). For hydrogen-bond motifs, see: Bernstein *et al.* (1995 ▶). For similar structures, see: El-Khouly *et al.* (2012 ▶); Öztürk Yildirim *et al.* (2012 ▶, 2013 ▶); Gündüz *et al.* (2012 ▶).
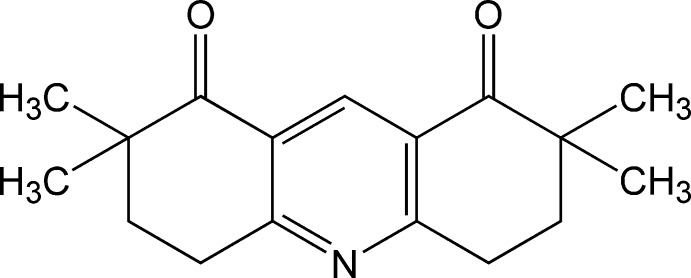


## Experimental

### 

#### Crystal data

C_17_H_21_NO_2_
*M* *_r_* = 271.35
Tetragonal, 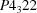

*a* = 9.99077 (19) Å
*c* = 14.5063 (4) Å
*V* = 1447.95 (6) Å^3^
*Z* = 4
Cu *K*α radiation
μ = 0.64 mm^−1^
*T* = 123 K
0.50 × 0.30 × 0.25 mm

#### Data collection

Agilent Xcalibur (Ruby, Gemini) diffractometer
Absorption correction: multi-scan [*CrysAlis RED* (Agilent, 2011 ▶), based on expressions derived by Clark & Reid (1995 ▶)] *T*_min_ = 0.740, *T*_max_ = 0.856
3055 measured reflections
1452 independent reflections
1349 reflections with *I* > 2σ(*I*)
*R*_int_ = 0.030

#### Refinement

*R*[*F* ^2^ > 2σ(*F* ^2^)] = 0.043
*wR*(*F* ^2^) = 0.122
*S* = 1.09
1452 reflections
94 parameters
H-atom parameters constrained
Δρ_max_ = 0.16 e Å^−3^
Δρ_min_ = −0.24 e Å^−3^

### 

Data collection: *CrysAlis PRO* (Agilent, 2011 ▶); cell refinement: *CrysAlis PRO*; data reduction: *CrysAlis PRO*; program(s) used to solve structure: *SHELXS97* (Sheldrick, 2008 ▶); program(s) used to refine structure: *SHELXL97* (Sheldrick, 2008 ▶); molecular graphics: *SHELXTL* (Sheldrick, 2008 ▶); software used to prepare material for publication: *SHELXTL*.

## Supplementary Material

Crystal structure: contains datablock(s) I, global. DOI: 10.1107/S1600536812048957/mw2098sup1.cif

Structure factors: contains datablock(s) I. DOI: 10.1107/S1600536812048957/mw2098Isup2.hkl

Supplementary material file. DOI: 10.1107/S1600536812048957/mw2098Isup3.cml

Additional supplementary materials:  crystallographic information; 3D view; checkCIF report

## Figures and Tables

**Table 1:** Hydrogen-bond geometry (Å, °)

*D*—H⋯*A*	*D*—H	H⋯*A*	*D*⋯*A*	*D*—H⋯*A*
C2—H2*B*⋯O1^i^	0.99	2.52	3.415 (2)	151

Symmetry code: (i) 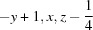.

**Fig. 1.**
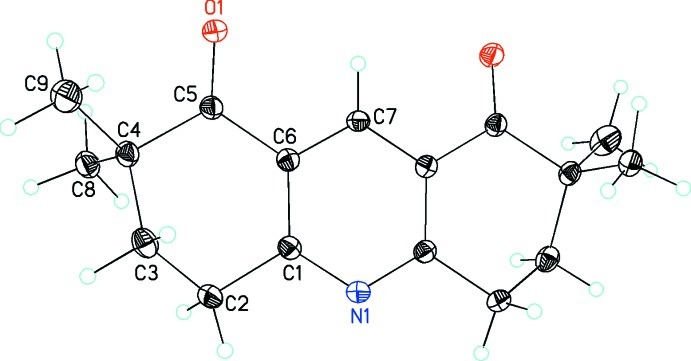
The molecular structure of (I), showing 30% probability displacement ellipsoids and the atom-numbering scheme. H atoms are drawn as spheres of arbitrary radii. Unlabelled atoms are related to labelled counterparts by the two-fold axis

**Fig. 2.**
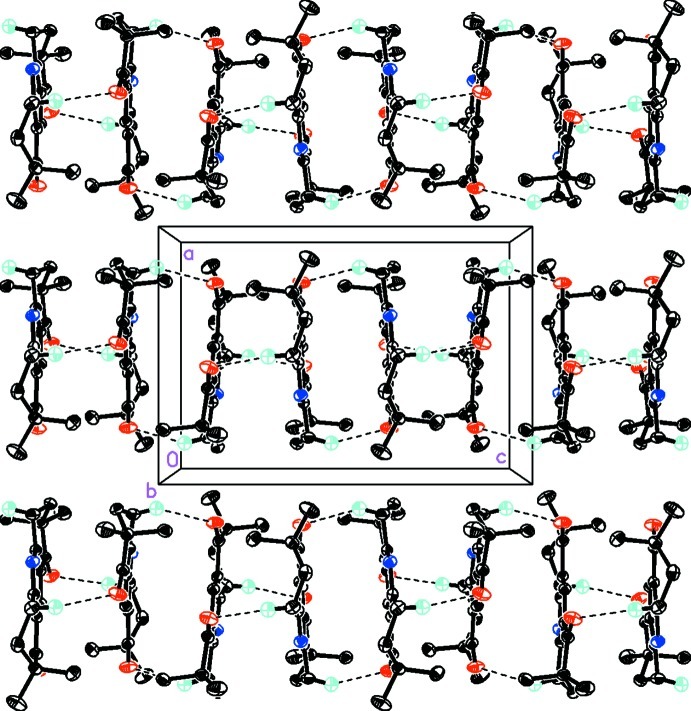
Crystal packing of (I) viewed along the a axis showing the three dimensional network. Dashed lines indicate the C—H···O interactions.

## supplementary crystallographic information

### Comment

Potassium channels play an important role in cell function in both excitable and non-excitable cells. Potassium channel openers, which open vascular potassium channels, have the potential to restrain or prevent contractile responses to excitatory stimuli or clamp the vessel in a relaxed condition. Their vasorelaxant effect is due to an increase in the potassium efflux through opening plasmalemmal potassium channels, which reduce calcium release from intracellular sources (Horiuchi *et al.*, 2001; Crestanello *et al.*, 2000). It is well known that 1,4-dihydropyridine (DHP) and its bicyclo (quinoline) and tricyclo (acridine) analogs are a well known group of calcium channel blockers that are established in the clinic as having vasodilator and anti-hypertensive functions. Potassium channel opener activities of these compounds are well known (Simşek *et al.*, 2004; Fincan *et al.*, 2012; Gündüz *et al.*, 2009; Pyrko, 2008; Li *et al.*, 2010).

The molecular structure of (I) is shown in Fig. 1. The asymmetric unit consists of one half of the molecule and the complete molecule is generated from the asymmetric unit by a twofold axis which passes through the N1 and C7 atoms. The keto bond distance (C5—O1) is 1.215 (2) Å and is comparable with those in similar structures obtained from the Cambrige Crystallographic Database (Allen, 2002).The deviation of atom C3 from the mean plane passing through C1, C2, C4, C5, C6 is 0.595 (2) Å. The dihedral angle between the mean planes of C1, C2, C5 and C6 and C1^i^, C2^i^, C5^i^ and C6^i^ (related by 2-fold axis) is 6.02 (3)°. The π conjugation along N1/C1/C6/C7/C6^i^/C1^i^ [N1—C1 = 1.3423 (18) Å, N1—C1^i^ = 1.3423 (18) Å, C1—C6 = 1.409 (2) Å, C6—C7 = 1.3861 (17) Å, C7—C6^i^ = 1.3861 (17) Å and C1^i^—C6^i^ = 1.409 (2) Å, symmetry code: (i) = *y*, *x*, -*z* + 5/4] indicates the strong aromaticity in the central ring, which makes all the atoms of the ring lie almost in a plane with the maximum deviation being -0.017 (1) Å for C1. This planarity of the central ring is further supported by the zero value for the puckering amplitude of this ring (Cremer & Pople, 1975). The unique cyclohexene ring (C1–C6) is in a sofa conformation with puckering parameters (Cremer & Pople, 1975) of Q_T_ = 0.435 (2) Å, θ = 48.8 (2)° and φ = 123.7 (3)°, respectively. The values of the bond lengths and bond angles are comparable with those of the related structures previously reported (El-Khouly *et al.*, 2012; Öztürk Yildirim *et al.*, 2012, 2013; Gündüz, *et al.*, 2012).

Molecules of (I) are linked to each other *via* weak intermolecular C—H···O hydrogen bonds forming D motifs (Bernstein *et al.*, 1995) as chains parallel to the *a* axis (Table 1, Fig. 2). In the crystal, weak π-π stacking interactions also contribute to the stabilization: [*Cg*1···*Cg*1^ii^ (symmetry code: (ii) = 1 - *y*, *x*, -1/4 + *z*) = 3.844 (7) Å; where *Cg*1 is the centroid of the N1/C1/C6/C7/C6^i^/C1^i^ (symmetry code: (i) = *y*, *x*, -*z* + 5/4) ring].

### Experimental

A mixture of paraformaldehyde (1.0 mmol), 4,4-dimethyl-1,3-cyclohexanedione (2.0 mmol) and 1 mL of glacial acetic acid was refluxed in 5 mL of methanol for 8 h. Ammonium acetate (5.0 mmol) was then added and reflux was continued until the reaction was completed (monitored by TLC). The mixture was evaporated under reduced pressure, the residue was treated with 5 mL of water and 20 mL of dichloromethane. The dichloromethane extract was dried over sodium sulfate and evaporated to give the desired product. Pure crystals suitable for X-ray structure analysis were obtained by slow evaporation method using methanol as a solvent.

### Refinement

H atoms bonded to C atoms were positioned geometrically and treated as riding with C—H = 0.95–0.99 Å and *U*_iso_(H) = 1.2*U*_eq_(C) for H, and C—H = 0.98 Å and *U*_iso_(H) = 1.5*U*_eq_(C) for methyl H. The crystal is a racemic twin with a BASF value of 0.3 (4).

### Crystal data

**Table:**

C_17_H_21_NO_2_	*D*_x_ = 1.245 Mg m^−^^3^
*M**_r_* = 271.35	Cu *K*α radiation, λ = 1.54184 Å
Tetragonal, *P*4_3_22	Cell parameters from 1551 reflections
Hall symbol: P 4cw 2c	θ = 3.0–75.1°
*a* = 9.99077 (19) Å	µ = 0.64 mm^−^^1^
*c* = 14.5063 (4) Å	*T* = 123 K
*V* = 1447.95 (6) Å^3^	Block, colorless
*Z* = 4	0.50 × 0.30 × 0.25 mm
*F*(000) = 584	

### Data collection

**Table:**

Agilent Xcalibur (Ruby, Gemini) diffractometer	1452 independent reflections
Radiation source: Enhance (Cu) X-ray Source	1349 reflections with *I* > 2σ(*I*)
Graphite monochromator	*R*_int_ = 0.030
Detector resolution: 10.5081 pixels mm^-1^	θ_max_ = 75.3°, θ_min_ = 4.4°
ω scans	*h* = −11→12
Absorption correction: multi-scan [*CrysAlis RED* (Agilent, 2011), based on expressions derived by Clark & Reid (1995)]	*k* = −12→7
*T*_min_ = 0.740, *T*_max_ = 0.856	*l* = −12→18
3055 measured reflections	

### Refinement

**Table:**

Refinement on *F*^2^	Primary atom site location: structure-invariant direct methods
Least-squares matrix: full	Secondary atom site location: difference Fourier map
*R*[*F*^2^ > 2σ(*F*^2^)] = 0.043	Hydrogen site location: inferred from neighbouring sites
*wR*(*F*^2^) = 0.122	H-atom parameters constrained
*S* = 1.09	*w* = 1/[σ^2^(*F*_o_^2^) + (0.0749*P*)^2^ + 0.0669*P*] where *P* = (*F*_o_^2^ + 2*F*_c_^2^)/3
1452 reflections	(Δ/σ)_max_ < 0.001
94 parameters	Δρ_max_ = 0.16 e Å^−^^3^
0 restraints	Δρ_min_ = −0.24 e Å^−^^3^

### Special details

**Table:**

Experimental. Absorption correction: CrysAlis RED, (Agilent, 2011) Empirical absorption correction using spherical harmonics, implemented in SCALE3 ABSPACK scaling algorithm. (Clark & Reid, 1995).
Geometry. All e.s.d.'s (except the e.s.d. in the dihedral angle between two l.s. planes) are estimated using the full covariance matrix. The cell e.s.d.'s are taken into account individually in the estimation of e.s.d.'s in distances, angles and torsion angles; correlations between e.s.d.'s in cell parameters are only used when they are defined by crystal symmetry. An approximate (isotropic) treatment of cell e.s.d.'s is used for estimating e.s.d.'s involving l.s. planes.
Refinement. Refinement of *F*^2^ against ALL reflections. The weighted *R*-factor *wR* and goodness of fit *S* are based on *F*^2^, conventional *R*-factors *R* are based on *F*, with *F* set to zero for negative *F*^2^. The threshold expression of *F*^2^ > σ(*F*^2^) is used only for calculating *R*-factors(gt) *etc*. and is not relevant to the choice of reflections for refinement. *R*-factors based on *F*^2^ are statistically about twice as large as those based on *F*, and *R*- factors based on ALL data will be even larger.

### Fractional atomic coordinates and isotropic or equivalent isotropic displacement parameters (Å^2^)

**Table:**

	*x*	*y*	*z*	*U*_iso_*/*U*_eq_	
O1	0.54930 (12)	0.19920 (12)	0.62530 (9)	0.0345 (3)	
N1	0.66208 (13)	0.66208 (13)	0.6250	0.0259 (4)	
C1	0.69529 (15)	0.53223 (15)	0.61859 (10)	0.0231 (3)	
C2	0.84076 (15)	0.49870 (18)	0.60647 (12)	0.0288 (4)	
H2A	0.8957	0.5649	0.6403	0.035*	
H2B	0.8643	0.5049	0.5403	0.035*	
C3	0.87361 (17)	0.35818 (18)	0.64155 (11)	0.0297 (4)	
H3A	0.8671	0.3579	0.7096	0.036*	
H3B	0.9674	0.3368	0.6251	0.036*	
C4	0.78234 (16)	0.24795 (17)	0.60323 (11)	0.0254 (4)	
C5	0.63570 (16)	0.28413 (16)	0.61821 (11)	0.0240 (3)	
C6	0.59904 (15)	0.42923 (15)	0.62063 (10)	0.0218 (3)	
C7	0.46519 (15)	0.46519 (15)	0.6250	0.0223 (4)	
H7A	0.3980	0.3980	0.6250	0.027*	
C8	0.79805 (17)	0.23165 (17)	0.49808 (12)	0.0319 (4)	
H8A	0.7307	0.1685	0.4752	0.048*	
H8B	0.8877	0.1974	0.4842	0.048*	
H8C	0.7856	0.3186	0.4680	0.048*	
C9	0.8130 (2)	0.1153 (2)	0.65133 (18)	0.0458 (6)	
H9A	0.7559	0.0448	0.6257	0.069*	
H9B	0.7955	0.1242	0.7175	0.069*	
H9C	0.9072	0.0920	0.6416	0.069*	

### Atomic displacement parameters (Å^2^)

**Table:**

	*U*^11^	*U*^22^	*U*^33^	*U*^12^	*U*^13^	*U*^23^
O1	0.0283 (6)	0.0225 (5)	0.0527 (8)	−0.0016 (5)	0.0096 (6)	0.0026 (6)
N1	0.0238 (6)	0.0238 (6)	0.0300 (9)	−0.0034 (7)	0.0020 (6)	−0.0020 (6)
C1	0.0220 (7)	0.0265 (8)	0.0209 (7)	−0.0006 (7)	0.0003 (6)	−0.0011 (6)
C2	0.0202 (7)	0.0309 (9)	0.0352 (8)	−0.0030 (6)	0.0014 (6)	−0.0046 (7)
C3	0.0222 (7)	0.0388 (9)	0.0283 (8)	0.0035 (7)	−0.0034 (7)	−0.0007 (7)
C4	0.0222 (8)	0.0251 (7)	0.0288 (8)	0.0040 (6)	0.0006 (6)	0.0038 (6)
C5	0.0233 (8)	0.0229 (8)	0.0257 (7)	0.0013 (6)	0.0033 (7)	0.0031 (6)
C6	0.0229 (7)	0.0227 (7)	0.0197 (7)	0.0001 (6)	0.0007 (6)	0.0020 (6)
C7	0.0213 (6)	0.0213 (6)	0.0243 (10)	−0.0026 (8)	−0.0004 (6)	0.0004 (6)
C8	0.0263 (8)	0.0357 (9)	0.0338 (9)	0.0008 (7)	0.0036 (7)	−0.0065 (8)
C9	0.0353 (10)	0.0389 (10)	0.0631 (13)	0.0080 (8)	−0.0002 (9)	0.0204 (10)

### Geometric parameters (Å, º)

**Table:**

O1—C5	1.215 (2)	C4—C9	1.528 (2)
N1—C1	1.3423 (18)	C4—C8	1.542 (2)
N1—C1^i^	1.3423 (18)	C5—C6	1.496 (2)
C1—C6	1.409 (2)	C6—C7	1.3861 (17)
C1—C2	1.502 (2)	C7—C6^i^	1.3861 (17)
C2—C3	1.529 (2)	C7—H7A	0.9500
C2—H2A	0.9900	C8—H8A	0.9800
C2—H2B	0.9900	C8—H8B	0.9800
C3—C4	1.534 (2)	C8—H8C	0.9800
C3—H3A	0.9900	C9—H9A	0.9800
C3—H3B	0.9900	C9—H9B	0.9800
C4—C5	1.525 (2)	C9—H9C	0.9800
			
C1—N1—C1^i^	118.85 (19)	O1—C5—C6	120.07 (15)
N1—C1—C6	122.40 (14)	O1—C5—C4	121.96 (15)
N1—C1—C2	117.57 (14)	C6—C5—C4	117.93 (13)
C6—C1—C2	120.01 (14)	C7—C6—C1	118.05 (15)
C1—C2—C3	111.93 (14)	C7—C6—C5	119.24 (14)
C1—C2—H2A	109.2	C1—C6—C5	122.71 (14)
C3—C2—H2A	109.2	C6^i^—C7—C6	120.1 (2)
C1—C2—H2B	109.2	C6^i^—C7—H7A	119.9
C3—C2—H2B	109.2	C6—C7—H7A	119.9
H2A—C2—H2B	107.9	C4—C8—H8A	109.5
C2—C3—C4	114.27 (13)	C4—C8—H8B	109.5
C2—C3—H3A	108.7	H8A—C8—H8B	109.5
C4—C3—H3A	108.7	C4—C8—H8C	109.5
C2—C3—H3B	108.7	H8A—C8—H8C	109.5
C4—C3—H3B	108.7	H8B—C8—H8C	109.5
H3A—C3—H3B	107.6	C4—C9—H9A	109.5
C5—C4—C9	109.45 (15)	C4—C9—H9B	109.5
C5—C4—C3	110.45 (13)	H9A—C9—H9B	109.5
C9—C4—C3	109.74 (15)	C4—C9—H9C	109.5
C5—C4—C8	105.29 (13)	H9A—C9—H9C	109.5
C9—C4—C8	109.85 (16)	H9B—C9—H9C	109.5
C3—C4—C8	111.95 (14)		
			
C1^i^—N1—C1—C6	−1.60 (11)	C3—C4—C5—C6	29.2 (2)
C1^i^—N1—C1—C2	176.83 (16)	C8—C4—C5—C6	−91.81 (16)
N1—C1—C2—C3	154.89 (12)	N1—C1—C6—C7	3.1 (2)
C6—C1—C2—C3	−26.6 (2)	C2—C1—C6—C7	−175.25 (13)
C1—C2—C3—C4	51.52 (19)	N1—C1—C6—C5	−176.91 (12)
C2—C3—C4—C5	−52.49 (18)	C2—C1—C6—C5	4.7 (2)
C2—C3—C4—C9	−173.25 (15)	O1—C5—C6—C7	−4.2 (2)
C2—C3—C4—C8	64.49 (18)	C4—C5—C6—C7	173.69 (12)
C9—C4—C5—O1	−32.0 (2)	O1—C5—C6—C1	175.89 (15)
C3—C4—C5—O1	−152.98 (16)	C4—C5—C6—C1	−6.3 (2)
C8—C4—C5—O1	85.99 (19)	C1—C6—C7—C6^i^	−1.48 (10)
C9—C4—C5—C6	150.15 (17)	C5—C6—C7—C6^i^	178.57 (16)

Symmetry code: (i) *y*, *x*, −*z*+5/4.

### Hydrogen-bond geometry (Å, º)

**Table:**

*D*—H···*A*	*D*—H	H···*A*	*D*···*A*	*D*—H···*A*
C2—H2*B*···O1^ii^	0.99	2.52	3.415 (2)	151

Symmetry code: (ii) −*y*+1, *x*, *z*−1/4.
